# A new species of the genus *Takydromus* (Squamata, Lacertidae) from southwestern Guangdong, China

**DOI:** 10.3897/zookeys.871.35947

**Published:** 2019-08-12

**Authors:** Jian Wang, Zhi-Tong Lyu, Chen-Yu Yang, Yu-Long Li, Ying-Yong Wang

**Affiliations:** 1 State Key Laboratory of Biocontrol / The Museum of Biology, School of Life Sciences, Sun Yat-sen University, Guangzhou 510275, China

**Keywords:** grass lizard, southern China, species diversity, taxonomy, *Takydromus yunkaiensis* sp. nov.

## Abstract

A new species, *Takydromus yunkaiensis* J. Wang, Lyu, & Y.Y. Wang, **sp. nov.** is described based on a series of specimens collected from the Yunkaishan Nature Reserve located in the southern Yunkai Mountains, western Guangdong Province, China. The new species is a sister taxon to *T. intermedius* with a genetic divergence of 8.0–8.5% in the mitochondrial cytochrome b gene, and differs from all known congeners by a combination of the following morphological characters: (1) body size moderate, SVL 37.8–56.0 mm in males, 42.6–60.8 mm in females; (2) dorsal ground color brown; ventral surface green to yellow-green, but light blue-green on chin and throat, posteriorly green in adult males; (3) dorsolateral lines paired, strikingly yellowish-white bordered by black above and below, invisible or indistinct in juveniles and adult females; (4) flanks of body blackish brown with light brown marks in adult males; (5) presence of four pairs of chin-shields; (6) four supraoculars on each side; (7) presence of a row of supracilary granules that separate supracilaries from supraoculars; (8) two postnasals; (9) enlarged dorsal scales in six longitudinal rows on trunk of body, with strong keel; (10) enlarged ventral scales in six longitudinal rows, strongly keeled in males, smooth but outermost rows weakly keeled in females; (11) enlarged and keeled lateral scales in a row above ventrals; (12) femoral pores 2–3 on each side; (13) subdigital lamellae 20–23 under the fourth finger, 23–30 under the fourth toe; and (14) the first 2–3 subdigital lamellae under the fourth toe divided. The discovery of *Takydromus yunkaiensis***sp. nov.** brings the total number of species of this genus to 24, of which nine occur in mainland China.

## Introduction

The Asian grass lizard genus *Takydromus* Daudin, 1802 currently contains 23 recognized species, widely distributed in the East Asian islands (Ryukyu Archipelago, Taiwan) and recorded from the Russian far east, extending southward across the Chinese mainland, Indochina, northeastern India, Borneo, the Natuna Islands, Sumatra, Bangka, and Java (Wang et al. 2017; Uetz et al. 2019). Eight species are recorded in mainland China: *T. albomaculosus* Wang, Gong, Liu & Wang, 2017, *T. amurensis* Peters, 1881, *T. intermedius* Stejneger, 1924, *T. kuehnei* van Denburgh, 1909, *T. septentrionalis* Günther, 1864, *T. sexlineatus* Daudin, 1802, *T. sylvaticus* Pope, 1928, and *T. wolteri* Fischer, 1885 (Zhao et al. 1999; Cai et al. 2011; Wang et al. 2017). In addition, *T. formosanus* Boulenger, 1894, *T. hsuehshanensis* Lin & Cheng, 1981, *T. luyeanus* Lue & Lin, 2008, *T. sauteri* Van Dengurgh, 1909, *T. stejnegeri* van Denburgh, 1912, and *T. viridipunctatus* Lue & Lin, 2008 are endemic to Taiwan Island; *T. dorsalis* Stejneger, 1904, *T. smaragdinus* Boulenger, 1887, *T. tachydromoides* Schlegel, 1838, and *T. toyamai* Takeda & Ota, 1996 are known only from Japan. Finally, *T. hani* Chou, Nguyen & Pauwels, 2001 and *T. madaensis* Bobrov, 2013 are only recorded from Vietnam while *T. khasiensis* Boulenger, 1917 and *T. sikkimensis* Günther, 1888 are only recorded from India.

**Figure 1.**
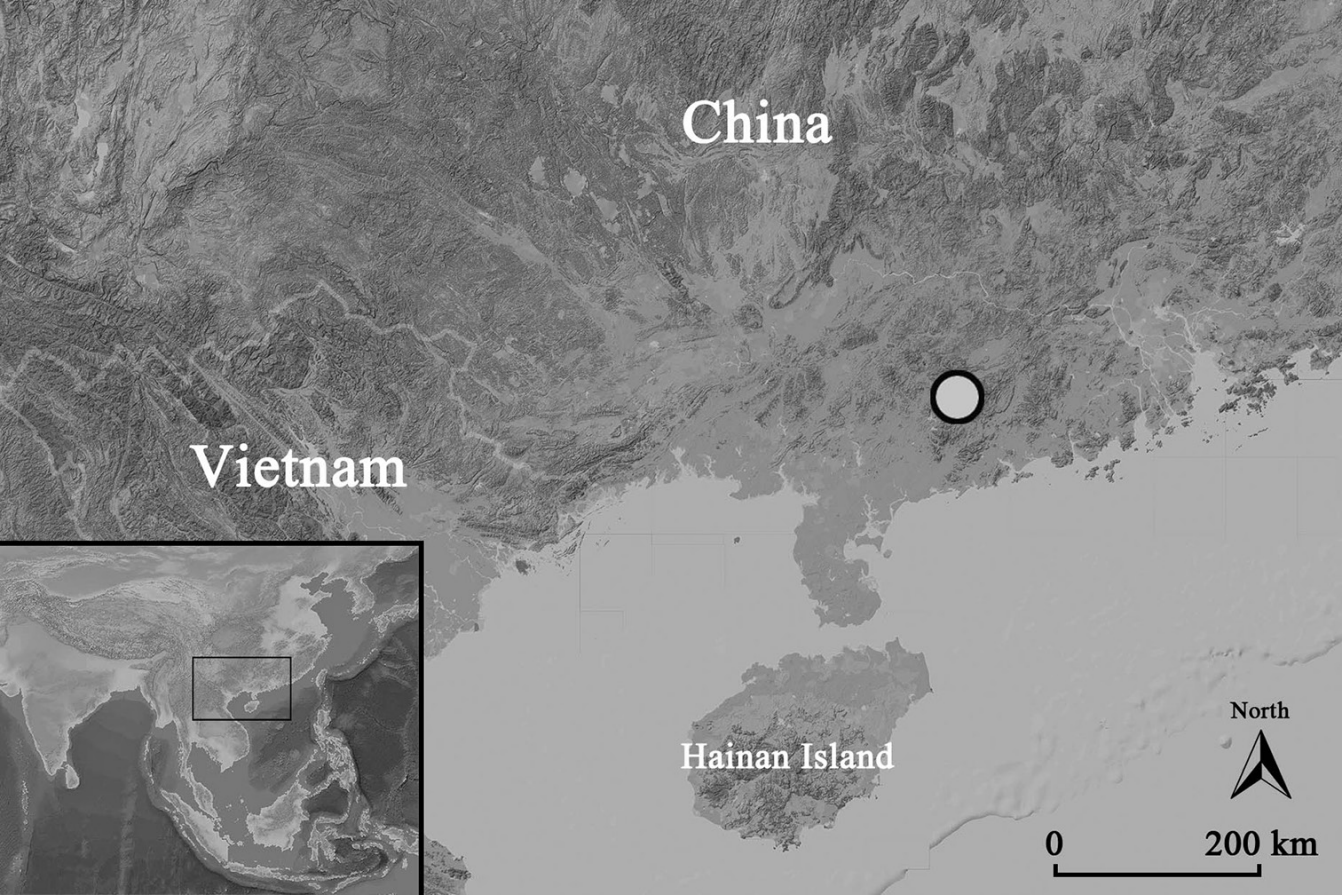
The type locality of *Takydromus yunkaiensis* sp. nov., Yunkaishan Nature Reserve.

Previous studies have revealed the very high biodiversity level of the genus *Takydromus* in southern China, for which the species diversity is just below that of Taiwan Island (Lue and Lin 2008; Wang et al. 2017). During repeated field surveys in the Yunkai Mountains, located in southwestern Guangdong Province (Fig. 1), a number of lacertid specimens were collected that could be assigned to the genus *Takydromus* by a combination of diagnostic characters defined by Arnold et al. (2007) and Zhao et al. (1999): (1) body slender with an extra-long tail, tail length usually more than two times larger than snout-vent length, (2) dorsal scales enlarged and keeled, ventral scales enlarged, keeled or smooth, (3) scales on flanks small and granular, (4) lateral teeth tricuspid, (5) temporal scales usually keeled, (6) 0–5 femoral pores on each side. Close examination of the external morphology and subsequent molecular analyses revealed that these specimens from Yunkaishan Nature Reserve, Guangdong Province, represented a distinct taxon. They are described below as a new species.

## Materials and methods

### Sampling

Samples sequenced for molecular analyses were obtained from two specimens of the undescribed *Takydromus* species from Yunkaishan Nature Reserve, Guangdong Province; the paratype specimen (SYS r001292) of *T. albomaculosus*; two specimens of *T. amurensis*; four specimens of *T. intermedius* including a topotypic specimen (SYS r001602) from Mt. Emei, Sichuan; four specimens of *T. kuehnei*; three specimens of *T. septentrionalis*; two specimens of *T. sexlineatus*; one specimen of *T. sylvaticus*; and two specimens of *T. wolteri*, all freshly collected from China. Additional 14 sequences of *T. dorsalis*, *T. formosanus*, *T. hsuehshanensis*, *T. sauteri*, *T. smaragdinus*, *T. stejnegeri*, *T. tachydromoides*, and *T. toyamai* were obtained from GenBank and three sequences of *Eremias persica* Blanford, 1875, *E. strauchi* Kessler, 1878, and *E. velox* (Pallas, 1771) also from GenBank were used as the out-groups. Details of samples sequenced for mitochondrial cytochrome b gene and their associated GenBank accession numbers are listed in Table 1.

**Table 1.** Localities, voucher information, and GenBank accession numbers (mitochondrial cytochrome b gene) for all specimens/sequences used in this study.

ID	Species	Locality	Voucher	GenBank Number
**1**	*Takydromus yunkaiensis* sp. nov.	China: Guangdong: Gaozhou: Xianrendong Scenic Area	SYS r001513	MN239954
**2**	*Takydromus yunkaiensis* sp. nov.	China: Guangdong: Gaozhou: Xianrendong Scenic Area	SYS r001514	MN239955
**3**	*Takydromus albomaculosus*	China: Guangdong: Ruyuan: Tianjingshan Forestry Station	SYS r001292 (paratype)	MF631870
**4**	*Takydromus amurensis*	China: Liaoning: Fushun: Nanzamu County: Mt. Langya	SYS r001890	MN239956
**5**	*Takydromus amurensis*	China: Liaoning: Fushun: Nanzamu County: Mt. Langya	SYS r001891	MN239957
**6**	*Takydromus dorsalis*	Japan	–	AY248460
**7**	*Takydromus dorsalis*	Japan	–	AY248461
**8**	*Takydromus formosanus*	China: Taiwan Island	–	AY248458
**9**	*Takydromus formosanus*	China: Taiwan Island	–	AY248459
**10**	*Takydromus hsuehshanensis*	China: Taiwan Island	–	AY248482
**11**	*Takydromus hsuehshanensis*	China: Taiwan Island	–	AY248483
**12**	*Takydromus intermedius*	China: Sichuan: Mt. Emei (type locality)	SYS r001602 (topotype)	MN239958
**13**	*Takydromus intermedius*	China: Guizhou: Libo: Maolan Nature Reserve	SYS r000856	MN239959
**14**	*Takydromus intermedius*	China: Guangxi: Hechi: Jiuwanshan Nature Reserve	SYS r001553	MN239960
**15**	*Takydromus intermedius*	China: Guangxi: Hechi: Cenwanglaoshan Nature Reserve	SYS r001741	MN239961
**16**	*Takydromus kuehnei*	China: Taiwan Island: Xinzhu County	SYS r001797	MN239962
**17**	*Takydromus kuehnei*	China: Taiwan Island: Taipei	SYS r001798	MN239963
**18**	*Takydromus kuehnei*	China: Jiangxi: Longnan: Jiulianshan Nature Reserve	SYS r001268	MN239964
**19**	*Takydromus kuehnei*	China: Zhaoqing: Fengkai: Heishiding Nature Reserve	SYS r001338	MN239965
**20**	*Takydromus sauteri*	China: Taiwan Island	–	AY248465
**21**	*Takydromus sauteri*	China: Taiwan Island	–	AY248466
**22**	*Takydromus septentrionalis*	China: Zhejiang: Lishui: Jingning County: Makeng Village	SYS r000912	MN239966
**23**	*Takydromus septentrionalis*	China: Zhejiang: Wenzhou: Chashan County	SYSr001886	MN239967
**24**	*Takydromus septentrionalis*	China: Jiangsu: Xiaotangshan	SYSr001882	MN239968
**25**	*Takydromus sexlineatus*	China: Zhaoqing: Fengkai: Heishiding Nature Reserve	SYS r001335	MN239969
**26**	*Takydromus sexlineatus*	China: Zhaoqing: Fengkai: Heishiding Nature Reserve	SYS r001336	MN239970
**27**	*Takydromus smaragdinus*	Japan: Akashima	–	LC066078
**28**	*Takydromus stejnegeri*	China: Taiwan Island	–	AY248473
**29**	*Takydromus stejnegeri*	China: Taiwan Island	–	AY248474
**30**	*Takydromus sylvaticus*	China: Fujian: Shaowu: Longhu Forestry Station	SYS r001276	MN239971
**31**	*Takydromus tachydromoides*	Japan: Nagasaki	– (topotype)	LC066067
**32**	*Takydromus tachydromoides*	Japan: Nagasaki	– (topotype)	LC066068
**33**	*Takydromus toyamai*	Japan	–	AY248480
**34**	*Takydromus wolteri*	China: Anhui: Mt. Langya	SYSr001888	MN239972
**35**	*Takydromus wolteri*	China: Anhui: Mt. Langya	SYSr001889	MN239973
**36**	*Eremias persica*	Iran	–	FJ416286
**37**	*Eremias strauchi*	Iran: Yengeje: Neyshabur-Khorasan Razavi	–	KJ468076
**38**	*Eremias velox*	Iran: Jajarm area-Northern Khorasan	–	KJ468081

All specimens were fixed in 10 % buffered formalin and later transferred to 70% ethanol for preservation, and deposited at the Museum of Biology, Sun Yat-sen University (**SYS**); liver tissue samples were separately preserved in 95% ethanol for molecular studies.

### DNA Extraction, PCR and sequencing

DNA was extracted from liver tissue using a standard phenol-chloroform extraction protocol (Sambrook et al. 1989). The fragment of mitochondrial cytochrome b gene was PCR amplified and sequenced using the primers L14919 5’-AACCACCGTTGTTATTCAACT-3’ and H16064 5’-CTTTGGTTTACAAGAACAATGCTTTA-3’ (Burbrink et al. 2000). PCR amplifications were performed in a 20 µL reaction volume with the following cycling conditions: an initial denaturing step at 95 °C for five min.; 35 cycles of denaturing at 95 °C for 40 s, annealing at 53 °C for 40 s, and extending at 72 °C for one min., and a final extending step of 72 °C for 10 min. PCR products were purified with spin columns. The purified products were sequenced with both forward and reverse primers using a BigDye Terminator Cycle Sequencing Kit (ThermoFisher Scientific, Waltham, MA) according to the manufacturer’s guidelines. The products were sequenced on an ABI Prism 3730 automated DNA sequencer (Shanghai Majorbio Bio-pharm Technology Co., Ltd).

### Phylogenetic analyses

Sequence alignments were first conducted using Clustal X 2.0 (Thompson et al. 1997), with default parameters and the alignment being checked and manually revised, if necessary. The data were tested in jmodeltest v2.1.2 with Akaike and Bayesian information criteria, resulting in the best-fitting nucleotide substitution models of GTR + I + G. Phylogenetic relationships were reconstructed using Maximum Likelihood (ML) as implemented in RaxmlGUI 1.3 (Silvestro and Michalak 2012), and Bayesian Inference (BI) using MrBayes 3.12 (Ronquist et al. 2012). For ML analysis, we used the rapid-bootstrapping algorithm (1000 replicates) with the thorough ML search option. Bootstrap values less than 60 were collapsed. For BI analysis, two independent runs with four Markov Chain Monte Carlo simulations were performed for ten million iterations and sampled every 1000^th^ iteration. The first 25 % of samples were discarded as burn-in. Convergence of the Markov Chain Monte Carlo simulations was assessed using Tracer v.1.4 (http://tree.bio.ed.ac.uk/software/tracer/). We also calculated pairwise sequence divergence based on uncorrected p-distance implemented in MEGA 6 (Tamura et al. 2013).

### Morphometrics

Measurements of all specimens were taken with a digital caliper to the nearest 0.1 mm. Abbreviations of measurements followed the convention of Lue and Lin (2008):

**ALL** arm-leg length (from insertion of the forelimb to insertion of hindlimb);

**HH** head height (measured at the highest point);

**HL** head length (from tip of snout to anterior margin of ear opening with claw);

**HLL** hindlimb length (from groin to tip of fourth toe);

**HW** head width (measured at the broadest point);

**LTL** length of fourth toe excluding claw;

**RUL** radius-ulna length;

**SAL** snout-arm length (from tip of snout to anterior insertion margin of forelimb);

**SEL** snout-eye length (from tip of snout to anterior margin of eye);

**SKL** skull length (from tip of snout to posterior margin of occipital);

**SVL** snout-vent length (from tip of snout to anterior margin of cloaca);

**TaL** tail length (from cloaca to tip of tail);

**TFL** tibia-fibula length.

Moreover, 20 external morphological characters were examined from the specimens listed in Appendix 1. Modified abbreviations of these characters followed Arnold (1997), Lue and Lin (2008) and Wang et al. (2017) as follows:

**ADSR** anterior dorsal scale rows, distinctly enlarged and keeled scales on anterior dorsum, counted transversely at position of forelimbs;

**CS** chin-shields;

**CSR** caudal scale rows, counted around the tail in the position of the 11^th^ to 13^th^ subcaudal scales;

**ESRF** enlarged and keeled lateral scales in longitudinal row(s) above ventrals on lower flanks;

**FP** femoral pores;

**IFL** infralabials;

**LDSN** dorsal scale numbers, counted longitudinally from posterior margin of occipital to posterior margin of hind limbs;

**MBSR** scales in a transverse row at mid-body, including ventrals;

**MDSR** transverse dorsal scale rows at mid-body;

**PDSR** posterior dorsal scale rows, counted transversely at the position of hind limbs;

**SDLF-IV** subdigital lamellae under fourth finger;

**SDLT-IV** subdigital lamellae under fourth toe;

**SPC** supraciliary;

**SPL** supralabials;

**SPO** supraocular;

**SPT** supratemporals;

**SSRF** small flat and granular scales in a transverse row on flank at mid-body;

**TSRF** enlarged and keeled scale rows above ventrals on flank;

**VN** ventral scale numbers, counted longitudinally from the posterior margin of collars to the anterior margin of precloacal scales;

**VR** ventral scale rows, counted transversely at mid-body.

Comparative morphological data were obtained from the literature for *Takydromus albomaculosus* (Wang et al. 2017), *T. hani* (Chou et al. 2001), *T. viridipunctatus*, and *T. luyeanus* (Lue and Lin 2008), *T. sikkimensis* (Bhupathy et al. 2009), *T. madaensis* (Bobrov 2013), *T. sylvaticus* (Pope 1928, 1929; Yang and Wang 2010), *T. smaragdinus* and *T. toyamai* (Takeda and Ota 1996); *T. kuehnei* (van Denburgh 1909; Arnold 1997; Norval et al. 2012; Wang et al. 2017), *T. intermedius* (Stejneger 1924; Wang et al. 2017), *T. amurensis*, *T. dorsalis*, *T. formosanus*, *T. hsuehshanensis*, *T. sauteri*, *T. stejnegeri*, and *T. tachydromoides* (Takeda and Ota 1996; Lue and Lin 2008), *T. sexlineatus*, *T. wolteri*, *T. septentrionalis*, and *T. khasiensis* (Arnold 1997; Zhao et al. 1999). All examined specimens are listed in Appendix I.

## Results

**Figure 2.**
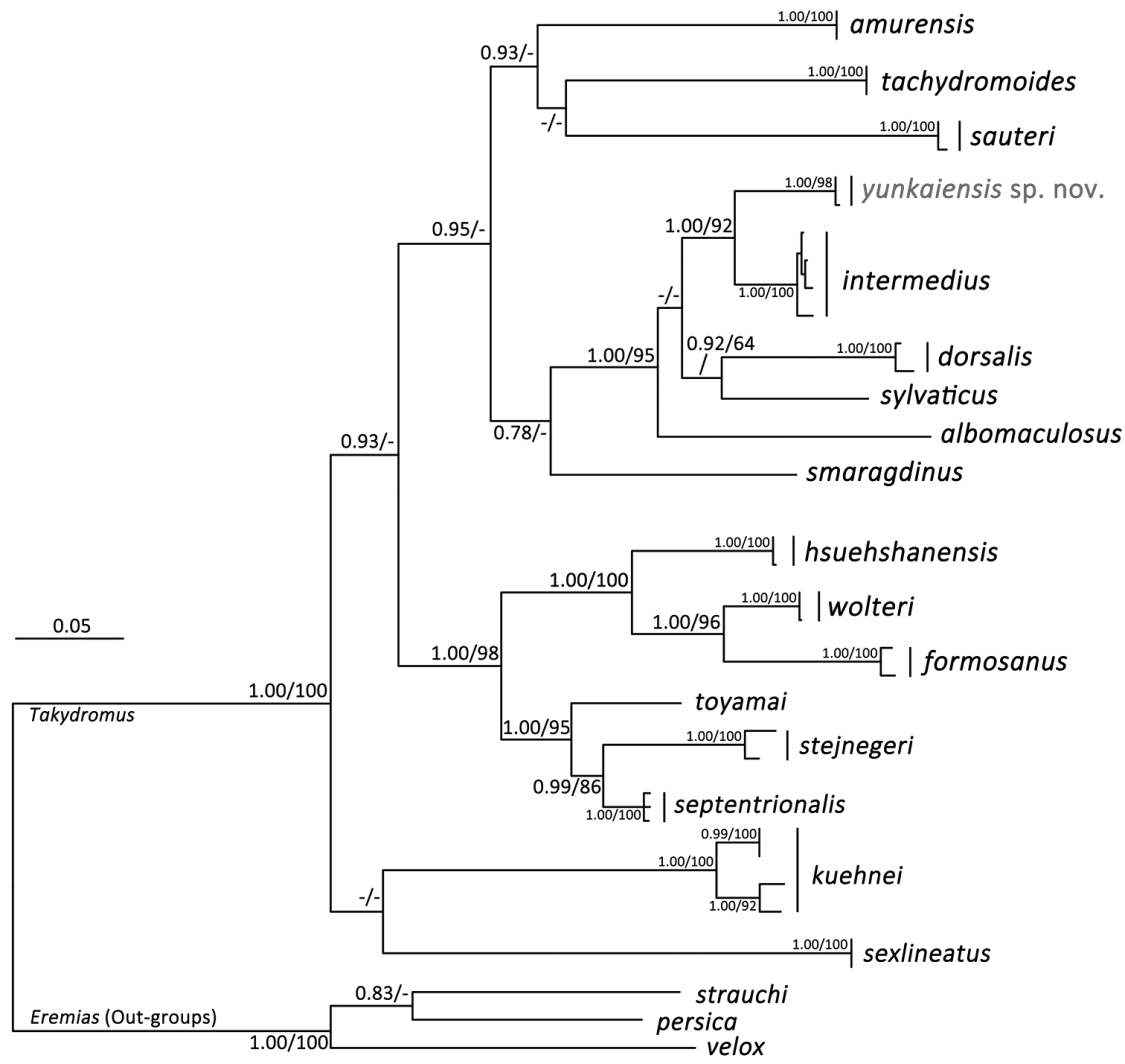
Bayesian Inference and Maximum Likelihood phylogenies. The Bayesian posterior probabilities (BPP) > 0.75 and the bootstrap supports for Maximum Likelihood analysis (BS) > 60 were retained.

BI and ML phylogenetic trees were constructed based on DNA sequences of the mitochondrial cytochrome b gene with a total length of 1074 -bp. The two analyses resulted in essentially identical topologies and are integrated in Figure 2, in which the Bayesian posterior probabilities (BPP) > 0.75 and the bootstrap supports (BS) for ML analysis > 60 were retained. The specimens from Yunkaishan Nature Reserve grouped in a strongly supported clade (BPP 1.00 and BS 98) with small divergence (*p*-distance 0.2 %), forming the sister taxon to *Takydromus intermedius* with strong support (BPP 1.00 and BS 92) and significant divergences (p-distance 8.0–8.5 %), and then to *T. dorsalis*, *T. sylvaticus*, and *T. albomaculosus* (BPP 1.00 and BS 95), indicating that the population from Yunkaishan Nature Reserve represents a separate evolutionary lineage.

Morphologically, the unnamed specimens can be clearly distinguished from its congeners by the following characters: (1) body size moderate, SVL 37.8–56.0 mm in males, 42.6–60.8 mm in females; (2) dorsal ground color brown; ventral surface green to yellow-green, but light blue-green on chin and throat, posteriorly green in adult males; (3) dorsolateral lines paired, strikingly yellowish-white bordered by black above and below, invisible or indistinct in juveniles and adult females; (4) flanks of body blackish brown with light brown marks in adult males; (5) the presence of four pairs of chin-shields; (6) four supraoculars on each side; (7) presence of a row of supracilary granules that separate supracilaries from supraoculars; (8) two postnasals; (9) enlarged dorsal scales in six longitudinal rows on trunk of body, with strong keel; (10) enlarged ventral scales in six longitudinal rows, strongly keeled in males, smooth but outermost rows weakly keeled in females; (11) enlarged and keeled lateral scales in a row above ventrals; (12) femoral pores 2–3 on each side; (13) subdigital lamellae 20–23 under the fourth finger, 23–30 under the fourth toe; and (14) the first 2–3 subdigital lamellae under the fourth toe divided.

Based on the comprehensive evidence of molecular and morphological analyses, we hereby describe these specimens from Yunkaishan Nature Reserve as a new species, *Takydromus yunkaiensis* sp. nov. Now, the genus *Takydromus* contains 24 species, nine of which are recorded from mainland China.

### Takydromus yunkaiensis

Taxon classificationAnimaliaSquamataLacertidae

J. Wang, Lyu & Y.Y. Wang sp. nov.

d27ac06f-78ed-513f-a4ed-0b12b96fa665

http://zoobank.org/E69D5272-696B-486C-AF44-3AB7C975A699

Fig. 3

#### Material.

**Holotype.** SYS r001580, adult male, collected by Jian Wang on 16 August 2016 from Dawuling Forestry Station (22°16'32.90"N, 111°11'42.87"E; 1500 m a.s.l.), Yunkaishan National Nature Reserve, Xinyi City, Guangdong Province, China.

**Paratypes.** Three adult males, collected by Ying-Yong Wang, Jian Wang, Zhi-Tong Lyu and Zhao-Chi Zeng: SYS r001439, 1442 on 15 and 16 April 2016, SYS r001684 on 17 April 2017, all from Dawuling Forestry Station (1200–1500 m a.s.l.). Six adult females: SYS r001513 and SYS r001514 collected by Jian Wang on 9 July 2016 from Xianrendong Scenic Area (22°165'45.99"N, 111°13'16.35"E; 1000 m a.s.l.), Yunkaishan National Nature Reserve, Xinyi City, Guangdong Province; SYS r001434 collected by Jian Wang and Zhi-Tong Lyu on 14 April 2016, SYS r001507 collected by Jian Wang on 28 June 2016, SYS r001581 collected by Jian Wang on 16 August 2016, and SYS r001901 collected by Jian Wang and Hong-Hui Chen on 10 April 2018, all from Dawuling Forestry Station (1200–1500 m a.s.l.).

**Figure 3.**
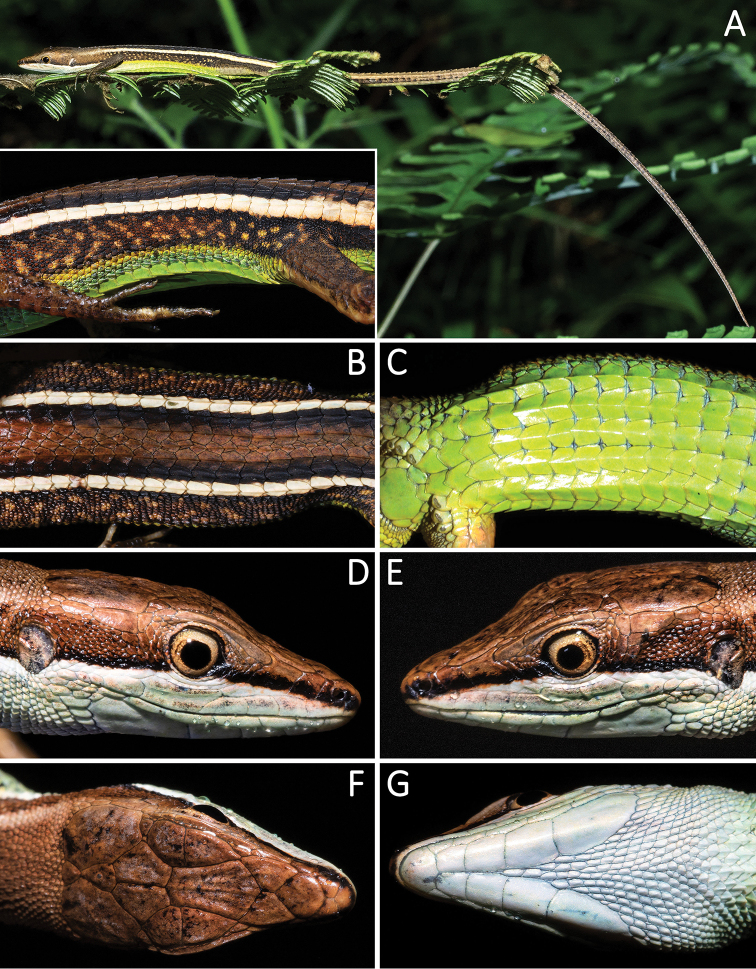
Morphological features of the adult male holotype SYS r001580 of *Takydromus yunkaiensis* sp. nov. in life. **A** Habitus view and close-up of flank **B** close-up of dorsal body **C** close-up of ventral body **D–G** close-up of head scales.

#### Etymology.

The specific epithet, *yunkaiensis*, is in reference to the type locality of the new species. We propose the standard name “Yunkai grass lizard” and the Chinese name “Yun Kai Cao Xi (云开草蜥)”.

#### Diagnosis.

(1) body size moderate, SVL 37.8–56.0 mm in males, 42.6–60.8 mm in females; (2) dorsal ground color brown; ventral surface green to yellow-green, but light blue-green on ventral head and neck, posteriorly green in adult males; (3) dorsolateral lines paired, strikingly yellowish-white bordered by black above and below, invisible or indistinct i n juveniles and adult females; (4) flanks of body blackish brown with light brown marks in adult males; (5) the presence of four pairs of chin-shields; (6) four supraoculars on each side; (7) presence of a row of supracilary granules that separate supracilaries from supraoculars; (8) two postnasals; (9) enlarged dorsal scales with strong keel in six longitudinal rows on trunk of body; (10) enlarged ventral scales in six longitudinal rows, strongly keeled in males, smooth but outermost rows weakly keeled in females; (11) enlarged and keeled lateral scales in a row above ventrals; (12) femoral pores 2–3 on each side; (13) subdigital lamellae 20–23 under the fourth finger, 23–30 under the fourth toe; and (14) the first 2–3 subdigital lamellae under the fourth toe divided.

#### Comparisons.

In this study we only compare the new species with the other 22 recognized species, excluding *Takydromus haughtonianus*, which is currently an uncertain species and poorly known (Jerdan 1870; Arnold 1997). Measurements, body proportions, and scale counts of the new species are listed in Tables 3 and 4; comparative data of the new species and nine other recognized members of the genus *Takydromus* occurring on the Chinese mainland are listed in Tables 5 and 6.

**Table 2.** Uncorrected *p*-distances among *Takydromus* species based on mitochondrial cytochrome b gene. *Takydromus yunkaiensis* sp. nov. **(1)–(2)**; *T. albomaculosus***(3)**; *T. amurensis***(4)–(5)**; *T. dorsalis***(6)–(7)**; *T. formosanus***(8)–(9)**; *T. hsuehshanensis***(10)–(11)**; *T. intermedius***(12)–(15)**; *T. kuehnei***(16)–(19)**; *T. sauteri***(20)–(21)**; *T. septentrionalis***(22)–(24**); *T. sexlineatus***(25)–(26)**; *T. smaragdinus***(27)**; *T. stejnegeri***(28)–(29)**; *T. sylvaticus***(30)**; *T. tachydromoides***(31)–(32)**; *T. toyamai***(33)**; *T. wolteri***(34)–(35)**.

SpecNo.	(1)–(2)	(3)	(4)–(5)	(6)–(7)	(8)–(9)	(10)–(11)	(12)–(15)	(16)–(19)	(20)–(21)	(22)–(24)	(25)–(26)	(27)	(28)–(29)	(30)	(31)–(32)	(33)	(34)–(35)
**(1)–(2)**	0.2																
**(3)**	16.5–17.0	–															
**(4)–(5)**	21.9–22.3	24.5	0														
**(6)–(7)**	13.5–14.4	18.1–18.2	23.2–23.6	1.2													
**(8)–(9)**	25.9–26.1	25.9–26.1	23.0–23.5	24.5–25.2	1.3												
**(10)–(11)**	21.9–22.2	23.4–23.6	23.6–23.8	22.6–23.3	15.8–16.2	0.1											
**(12)–(15)**	**8.0–8.5**	16.3–16.9	20.3–21.3	13.9–15.0	23.1–23.9	20.8–21.6	0.4–1.6										
**(16)–(19)**	21.5–22.8	24.2–25.2	25.6–25.9	23.0–24.2	22.3–23.6	20.2–22.5	24.2–25.4	0–5.4									
**(20)–(21)**	23.5	25.5–25.6	22.3–22.5	22.3–22.7	23.2–23.6	23.2–23.6	22.1–23.1	23.9–25.3	0.4								
**(22)–(24**)	21.1–22.1	24.3–24.7	20.2–22.2	24.2–24.8	17.8–20.0	15.8–17.5	20.2–21.3	21.4–23.4	24.1–24.8	0–0.6							
**(25)–(26)**	21.0–21.8	25.9	27.6	23.3	25.7–25.9	25.5–25.7	23.4–23.8	24.2–25.0	26.6–26.7	22.1–23.9	0						
**(27)**	18.9–19.7	21.7	19.8	19.3–19.7	24.2–24.6	21.2–21.4	19.0–19.4	23.4–24.0	21.0–21.2	22.9–23.7	26.3	-					
**(28)–(29)**	23.6–24.3	24.4–24.6	20.1–20.3	23.6–24.3	17.2–18.2	16.2–16.8	21.7–23.0	20.1–21.8	25.2–25.6	10.3–12.4	24.3–25.2	21.2–21.7	2.2				
**(30)**	13.3–13.4	18.5	21.5	13.4–13.8	25.6–25.8	20.1–20.3	12.5–12.8	22.2–23.8	22.7–22.9	19.9–20.6	23.7	18.7	20.6–21.2	-			
**(31)–(32)**	21.8–22.2	23.7	22.5	23.0–23.3	26.6–26.8	23.9–24.1	22.6–23.0	23.7–24.4	21.7	22.4–22.7	29.2	21.6	24.0–24.2	22.2	0		
**(33)**	21.7–21.9	25.2	19.6	21.5–21.9	19.1–19.9	15.7–15.9	20.9–21.9	22.2–23.2	22.7–23.2	9.5–10.9	21.6	21.9	11.7–12.6	20.5	22.8	/	
**(34)–(35)**	22.8–23.3	23.9–24.1	21.6	23.1–24.2	10.6–11.1	12.7–12.9	22.8–23.5	23.3–24.1	21.8–22.6	17.2–17.8	25.9–26.0	21.9–22.1	17.4–17.6	21.7–21.8	24.3–24.4	18.1	0.1

**Table 3.** Measurements and body proportions of type series of *Takydromus yunkaiensis* sp. nov.

Voucher	SYS r	SYS r	SYS r	SYS r	SYS r	SYS r	SYS r	SYS r	SYS r	SYS r
Number	001439	001442	001580	001684	001434	001507	001513	001514	001581	001901
**Sex**	♂	♂	♂	♂	♀	♀	♀	♀	♀	♀
**SVL**	37.8	42.1	43.0	56.0	42.6	60.8	52.5	47.6	49.9	51.9
**TaL**	100.4	112.0	111.3	155.0	111.5	155.3	143.5	75.7 (broken tail)	148.3	156.7
**HL**	9.6	10.5	11.1	14.8	10.4	16.3	13.8	13.0	12.0	13.8
**HW**	6.4	6.6	6.7	8.1	6.5	7.6	6.9	6.3	7.4	7.7
**HH**	4.6	5.1	5.2	6.0	5.3	6.3	5.3	5.1	5.3	5.5
**SKL**	10.1	10.9	11.0	14.5	11.0	15.6	12.0	11.3	12.5	13.3
**SEL**	4.6	4.8	5.0	6.4	4.3	6.8	5.4	5.4	5.6	6.3
**ALL**	17.7	19.8	19.7	28.3	21.3	30.9	25.0	23.4	25.3	26.6
**SAL**	14.6	16.6	18.0	21.6	17.7	23.9	20.0	18.5	19.3	20.0
**RUL**	4.1	5.6	5.6	6.5	4.6	6.9	5.9	5.8	6.5	6.0
**HLL**	20.0	25.2	25.4	28.3	21.7	29.4	28.1	24.4	28.4	26.9
**TFL**	4.9	6.3	6.3	7.5	5.3	8.4	7.9	5.9	8.0	7.4
**LTL**	5.3	7.7	7.9	10.0	7.2	10.2	9.3	8.4	9.2	9.4
**TaL/SVL**	2.66	2.66	2.59	2.77	2.62	2.55	2.73	1.59	2.97	3.02
**HL/SVL**	0.25	0.25	0.26	0.26	0.24	0.27	0.26	0.27	0.24	0.27
**HL/HW**	1.50	1.58	1.66	1.83	1.61	2.14	2.01	2.06	1.63	1.79
**SKL/HL**	1.05	1.04	0.99	0.98	1.06	0.96	0.87	0.87	1.04	0.96
**SEL/HL**	0.48	0.46	0.45	0.43	0.41	0.42	0.39	0.41	0.47	0.46
**ALL/SVL**	0.47	0.47	0.46	0.51	0.50	0.51	0.48	0.49	0.51	0.51
**SAL/SVL**	0.39	0.39	0.42	0.39	0.42	0.39	0.38	0.39	0.39	0.39
**RUL/SVL**	0.11	0.13	0.13	0.12	0.11	0.11	0.11	0.12	0.13	0.12
**HLL/SVL**	0.53	0.60	0.59	0.51	0.51	0.48	0.53	0.51	0.57	0.52
**TFL/SVL**	0.13	0.15	0.15	0.13	0.12	0.14	0.15	0.12	0.16	0.14
**LTL/SVL**	0.14	0.18	0.18	0.18	0.17	0.17	0.18	0.18	0.19	0.18
**HLL/ALL**	1.13	1.27	1.29	1.00	1.02	0.95	0.13	1.04	1.12	1.01

**Table 4.** Scale counts of type series of *Takydromus yunkaiensis* sp. nov.

Voucher	SYS r	SYS r	SYS r	SYS r	SYS r	SYS r	SYS r	SYS r	SYS r	SYS r
No.	001434	001439	001442	001507	001513	001514	001580	001581	001684	001901
**CS**	4	4	4	4	4	4	4	4	4	4
**FP**	3	3	3	3	2	2	3	3	3	3
**SPL**	7	7	7	7	7	7	6	7/6	7	6
**IFL**	7	7	7	7	7	6	6	6/7	7	6
**SPO**	4	4	4	4	4	4	4	4	4	4
**SPC**	4	4	4	3	4	4	4/2	4	4	4
**SPT**	3/4	4/3	4/3	4	4/3	4/3	3	3/4	3/4	3
**ADSR**	9	10	9	9	9	9	9	9	9	9
**PDSR**	7	7	7	7	7	7	7	7	7	7
**MDSR**	8	8	8	8	8	8	7	8	7	7
**LDSN**	47	49	51	47	49	47	47	51	47	47
**MBSR**	40	44	42	40	46	41	42	41	46	41
**SSRF**	13/13	17/13	12/16	13/13	16/16	13/14	14/15	14/13	17/16	14/14
**VR**	6	6	6	6	6	6	6	6	6	6
**VN**	25	26	25	25	26	25	24	26	25	27
**ESRF**	1	1	1	1	1	1	1	1	1	1
**CSR**	13	13	12	10	12	13	12	13	13	12
**SDLF-4**	20	20	22	23	23	21	21	23	20	21
**SDLT-4**	27	23	26	28	30	27	26	28	27	25

**Table 5.** Selected body proportions of *Takydromus yunkaiensis* sp. nov. (data of the female paratype SYS r001514 with a broken tail is not included), and its morphologically most similar species *T. intermedius* and *T. kuehnei*; data obtained from Wang et al. (2017).

T. spp.	*yunkaiensis* sp. nov.		* intermedius *		* kuehnei *	
Sex	♂ (N = 4)	♀ (N = 5)	♂ (N = 1)	♀ (N = 3)	♂ (N = 2)	♀ (N = 1)
**TaL/SVL**	2.59–2.77	2.55–3.02	2.22	2.54–3.25	3.07–3.08	-
	(2.67±0.07)	(2.78±0.21)		(2.90±0.36)	(3.08)	
**HL/SVL**	0.25–0.26	0.24–0.27	0.25	0.22–0.25	0.24–0.25	0.22
	(0.26±0.01)	(0.26±0.01)		(0.24±0.02)	(0.25)	
**HL/HW**	1.50–1.83	1.61–2.14	1.80	1.68–1.72	1.69–1.83	1.84
	(1.64±1.14)	(1.87±0.23)		(1.70±0.02)	(1.76)	
**SKL/HL**	0.98–1.05	0.87–1.06	1.04	1.02–1.05	1.02–1.03	1.05
	(1.01±0.04)	(0.96±0.08)		(1.04±0.02)	(1.02)	
**SEL/HL**	0.43–0.48	0.39–0.47	0.46	0.46–0.48	0.45–0.46	0.49
	(0.46±0.02)	(0.43±0.03)		(0.47±0.01)	(0.46)	
**ALL/SVL**	0.46–0.51	0.48–0.51	0.55	0.53–0.55	0.52–0.53	0.58
	(0.48±0.02)	(0.50±0.01)		(0.54±0.01)	(0.53)	
**SAL/SVL**	0.39–0.42	0.38–0.42	0.39	0.37–0.42	0.38–0.39	0.36
	(0.40±0.02)	(0.39±0.01)		(0.40±0.03)	(0.38)	
**RUL/SVL**	0.11–0.13	0.11–0.13	0.16	0.12–0.14	0.13–0.14	0.11
	(0.12±0.01)	(0.12±0.01)		(0.14±0.01)	(0.13)	
**HLL/SVL**	0.51–0.60	0.48–0.57	0.55	0.48–0.53	0.54–0.57	0.49
	(0.56±0.05)	(0.52±0.03)		(0.51±0.03)	(0.56)	
**TFL/SVL**	0.13–0.15	0.12–0.16	0.17	0.14–0.16	0.14–0.16	0.13
	(0.14±0.01)	(0.14±0.01)		(0.15±0.01)	(0.15)	
**LTL/SVL**	0.14–0.18	0.17–0.19	0.19	0.16–0.20	0.20	0.19
	(0.17±0.02)	(0.18±0.01)		(0.19±0.02)		
**HLL/ALL**	1.00–1.29	0.95–1.13	0.99	0.89–1.01	1.03–1.09	0.85
	(1.17±0.14)	(1.04±0.07)		(0.96±0.06)	(1.06)	

**Table 6.** Selected scale counts of the nine species of the genus *Takydromus* recorded from the Chinese mainland, modified from Wang et al. (2017); differences are marked in bold.

Takydromus	*yunkaiensis* sp. nov.	* albomaculosus *	* amurensis *	* wolteri *	* septentrionalis *	* sexlineatus *	* intermedius *	* kuehnei *	* sylvaticus *
Species	(N = 10)	(N = 2)	(N = 2)	(N = 1)	(N = 25)	(N = 5)	(N = 8)	(N = 5)	(N = 3)
**CS**	**4**	4	4	4	**3**	**3**	4–5	4 (rare 3*)	4
**FP**	**2–3**	3–4	**4**	**1**	**1**	**1**	2–3	3–5	3
**SPL**	6–7	6–7	5–7	7	5–8	5–6	6–7	6–7	5–7
**IFL**	**6–7**	6–7	6–7	6–7	5–6	**4–5**	5–7	5–6	5–7
**SPO**	**4**	3 (rare 4^#^)	4	4	4 (rare 3^■^)	**3**	4	4	4
**SPC**	4 (rarely 2, 3^▲^)	4–6	4	4	3–5	3	4–5	4	4–5
**SPT**	3–4	3	2–3	3	1–4	2–3	2–5	3–4	2–4
**ADSR**	**9–10**	**6**	**7–8**	9	**6**–8	**6**	**6–8**	**5–7**	/
**PDSR**	**7**	**6**	6–7	7	**4–6**	**4**	**6**	**6**	**9–10**
**MDSR**	**7–8**	7	7–8	8	**5–6**	**4**	7–8	6–7	**11–14**
**LDSN**	**47–51**	**52–53**	**46**	**56**	**37–46**	**34–35**	**36–46**	42–47	**67–81**
**MBSR**	**40–46**	42–43	**33–38**	**36**	34–42	**28–33**	40–44	39–44	45–47
**SSRF**	**12–17**	13–14	**5–9**	**10**	**7–11**	**6–8**	12–15	13–16	13
**VR**	**6**	6	**8**	**8**	**8**	**8**	6	6	6
**VN**	**24–27**	23–26	27	**30**	25–29	26–27	21–24	27–29	26–29
**ESRF**	**1**	1	1–3	**3**	**2–3**	**2–3**	1	0–1	**0**
**CSR**	**10–13**	12	**16–18**	**16**	12–14	**14**	12	12–13	12
**SDLF-4**	**20–23**	23–24	**18–19**	**17**	18–22	**13–16**	20–21	18–20	21–22
**SDLT-4**	**23–30**	29–30	24–25	22–23	23–28	**19**	26–27	23–24	27–28

^▲^: Two supraciliaries only present on right side of the holotype SYS r001580 and three present on both sides of SYS r001507, *Takydromus yunkaiensis* sp. nov.; *: Three chin-shields only present on left side of SYS r001338, *T. kuehnei*; **^#^**: Four supraoculars only present on right side of SYS r001292, *T. albomaculosus*; **^■^**: Three supraoculars only present on one side in three of 25 specimens of *T. septentrionalis*.

In our phylogenetic tree, *Takydromus yunkaiensis* sp. nov. is a sister taxon to *T. intermedius*, from which it differs by having two postnasals (only one in *T. intermedius*), having a pair of strikingly yellowish-white dorsolateral lines in adult males (vs. always absent or indistinct in *T. intermedius*), flanks of body blackish brown with light brown spots in adult males (vs. pure brown without spots in *T. intermedius*), ADSR 9–10, PDSR 7 (vs. ADSR 6–8, PDSR 6 in *T. intermedius*).

**Figure 4.**
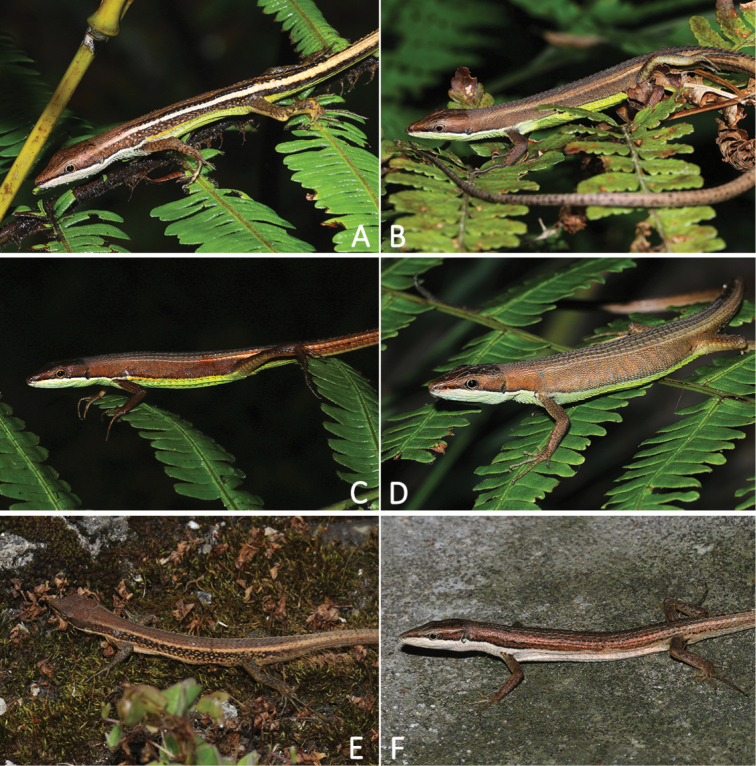
Sexual dimorphism in color patterns. **A** Male paratype of *Takydromus yunkaiensis* sp. nov. (SYS r001439) **B** female paratype of *T. yunkaiensis* sp. nov. (SYS r001901) **C** male topotype of *T. intermedius* (SYS r001601) from Mt. Emei, China **D** female topotype of *T. intermedius* (SYS r001602) from Mt. Emei, China **E** male *T. kuehnei* (SYS r001268) from Jiulianshan Nature Reserve, China **F** female topotype of *T. kuehnei* (SYS r001798) from Taiwan Island, China.

Morphologically, *Takydromus yunkaiensis* sp. nov. is most similar to *T. kuehnei* (Fig. 4). The new species can be distinguished from *T. kuehnei* by having a pair of strikingly yellowish-white dorsolateral lines in adult males (vs. absent or dorsolateral stripes blurred, pale brown only present in old individuals in *T. kuehnei*); surface of ventrals green (vs. surface of ventrals white or light yellow in *T. kuehnei*), ADSR 9–10, PDSR 7 (vs. ADSR 5–7, PDSR 6 in *T. kuehnei*); TaL/SVL 2.59–2.77 in males (vs. tail relatively longer, TaL/SVL 3.07–3.08 in *T. kuehnei*); relatively shorter trunk (arm-leg length), ALL/SVL 0.46–0.51 in males, 0.48–0.51 in females (vs. relatively larger arm-leg length, ALL/SVL 0.52–0.53 in males and 0.58 in female of *T. kuehnei*).

From the remaining six *Takydromus* species which occur on mainland China (*T. albomaculosus*, *T. amurensis*, *T. wolteri*, *T. septentrionalis*, *T. sexlineatus*, and *T. sylvaticus*), *Takydromus yunkaiensis* sp. nov. can be distinguished by having dense mottles on flanks in males (vs. several particular white round spots on the flanks in *T. albomaculosus*; white ocellus bordered by black edges in males of *T. sexlineatus*); dorsum brown (vs. dorsum green in *T. sylvaticus*); four pairs of chin-shields (vs. three in *T. septentrionalis* and *T. sexlineatus*); two or three pairs of femoral pores (vs. only one in *T. wolteri*, *T. septentrionalis* and *T. sexlineatus*; four in *T. amurensis*); IFL 6–7 (vs. 4–5 in *T. sexlineatus*); SPO 4 (vs. three in *T. sexlineatus*); ADSR 9–10 (vs. six in *T. albomaculosus* and *T. sexlineatus*; 7–8 in *T. amurensis*; 6–8 in *T. septentrionalis*); PDSR 7 (vs. six in *T. albomaculosus*; 4–6 in *T. septentrionalis*; four in *T. sexlineatus*; 9–10 in *T. sylvaticus*); MDSR 7–8 (vs. 5–6 in *T. septentrionalis*; 4 in *T. sexlineatus*; 11–14 in *T. sylvaticus*); LDSN 47–51 (vs. 56 in *T. wolteri*; 67–81 in *T. sylvaticus*); ESRF 1 (vs. three in *T. wolteri*; 2–3 in *T. septentrionalis* and *T. sexlineatus*; none in *T. sylvaticus*).

*Takydromus yunkaiensis* sp. nov. differs from *T. formosanus*, *T. hsuehshanensis*, *T. luyeanus*, *T. sauteri*, *T. stejnegeri*, and *T. viridipunctatus*, which only occurred in Taiwan Island of China, by having four pairs of chin-shields (vs. three pairs in *T. formosanus*, *T. viridipunctatus*, *T. luyeanus*, *T. hsuehshanensis* and *T. stejnegeri*); FP 2–3 pairs (vs. only one in *T. sauteri* and *T. stejnegeri*); VR 6 (vs. eight in *T. formosanus*, *T. stejnegeri* , *T. viridipunctatus* and *T. luyeanus*); ventrals keeled (vs. ventrals smooth in *T. hsuehshanensis*); mottles on flanks in males (vs. absent in males of *T. formosanus*, *T. sauteri* and *T. stejnegeri*); surface of ventrals green (vs. surface of ventrals white in *T. formosanus*, *T. hsuehshanensis*, and *T. sauteri*); rostral and nostril separated (vs. rostral touching nostril in *T. sauteri*); dorsum brown (vs. dorsum green in *T. sauteri*).

*Takydromus yunkaiensis* sp. nov. differs from *T. dorsalis*, *T. smaragdinus*, *T. tachydromoides*, and *T. toyamai*, which only occur in Japan, by having a brown dorsum (vs. green dorsum in *T. dorsalis*, *T. smaragdinus*, and *T. toyamai*); dorsal scales large, in longitudinal rows (vs. dorsal scales small, not in obvious longitudinal rows in *T. dorsalis*); FP 2–3 pairs (vs. only one in *T. smaragdinus* and *T. toyamai*); ventrals keeled (vs. smooth in *T. tachydromoides*); VR 6 (vs. 8 in *T. tachydromoides* and *T. toyamai*); CS 4 pairs (vs. 3 in *T. smaragdinus* and *T. toyamai*).

*Takydromus yunkaiensis* sp. nov. differs from the remaining four members, *T. hani* and *T. madaensis* from Vietnam, *T. khasiensis* and *T. sikkimensis* from India, by having the dorsum brown (vs. dorsum green in *T. hani*); VR 6 (vs. 8 in *T. hani* and *T. khasiensis*; VR 12 in *T. sikkimensis*); CS 4 pairs (vs. 3 in *T. khasiensis* and *T. sikkimensis*); FP 2–3 pairs (vs. FP 6–8 in *T. hani*); loreals 2 (vs. 3 in *T. madaensis*); SPO 4 (vs. 3 in *T. madaensis*); SDLT-4 23–30 (vs. SDLT-4 17 in *T. madaensis*).

#### Description of holotype.

Adult male. Body size slightly small, SVL 43.0 mm; trunk of body short, ALL 19.7 mm, 46 % of SVL; head slightly long, HL 11.1, HW 6.7 mm, HH 5.2 mm, HL 26 % of SVL; skull length larger than head length, SKL 11.9 mm; snout moderately long, SEL in 5.0 mm, SEL 45 % of HL. Rostral large, pentagonal, visible in dorsal view, in contact with the first supralabials posteriorly on both sides, and supranasals dorsolaterally; nostril surrounded by a supranasal, two postnasals and the first supralabial on each side; one supranasal on each side, large, in contact with each other dorso-medially, separating rostral from frontonasal, and in contact with the upper postnasal posteriorly, not in contact with the anterior loreal; postnasals two, both in contact with the anterior loreal posteriorly, the upper one in contact with supranasal dorsolaterally, with frontonasal dorsally, the lower one in contact with the first supralabial ventrally; supralabials six on each side, the fifth one largest, under the eye; two loreals on each side, anterior one smaller than posterior one; posterior loreal in contact with anteriormost supraocular and anteriormost supraciliary scale posteriorly; four supraoculars on each side, the posteriormost one much smaller than others; supraciliaries four on left side, the second one longest; supraciliaries two on right side, the first one longest; supracilary granules arranged in a row, separated supracilaries from supraoculars; frontonasal large, smooth, hexagonal, separated from frontal by a pair of prefrontals; prefrontals two, weakly keeled, in contact with each other medially, with frontal and anterior two supraoculars posteriorly, with loreals laterally, respectively; a single frontal hexagonal, weakly keeled, in contact with second and third supraoculars laterally, with frontoparietals posteriorly; frontoparietals two, pentagonal, in contact with each other medially, with parietal and interparietal posteriorly, respectively; interparietal diamond, surrounded by two frontoparietals, two parietals and the single occipital; parietal pit located in the central of interparietal, distinctly visible; parietals two, large, weakly keeled, slightly in contact with each other medially; a single occipital between two parietals; temporal scales granular, slightly keeled; supratemporals three on each side, keeled, anteriormost one largest, longer than total length of posterior two; mental large, semielliptical; infralabials six on each side; four pairs of chin-shields, anterior two pairs in contact with each other medially, posterior two pairs separated from each other by gular scales; following gular scales gradually increasing in size, keeled, and become imbricated; enlarged, strongly keeled median gular scales extending anteriorly to the line joined posterior edges of ears; collars clear, composed of scales in ten rows pointed backwards, and forming a free serration; enlarged, imbricated dorsal scales on body with strong keel oriented posteriorly that form continuous ridges, extending anteriorly beyond forelimbs on to the nape, in nine rows in position of forelimbs, seven rows in position of hindlimbs; seven rows at mid-body, including a much smaller and discontinuous central row; longitudinal dorsal scales (LDSN) 47; ventrals in six rows, imbricate, strongly keeled and pointed posteriorly; enlarged and keeled lateral scales in a row above ventrals; longitudinal ventral scales (VN) 24; small flat and granular scales in a transverse row on flank at mid-body (SSRF) 14 on left side and 15 on right side, including a row of scales (enlarged and keeled, shorter than ventrals) adjoining the ventrals; four rows of scales on lower flanks reduced, flattened, keeled; nine rows of small granular scales on upper flanks on left side and ten on right side; a discontinuous row of scales adjoining outermost dorsal scale row reduced, flattened, keeled; a total of 42 scales (MBSR) in a transverse row in mid-body region; a single precloacal entire, enlarged, surrounded by eight continuous moderately sized scales anteriorly and laterally; three femoral pores on each side.

Forelimbs moderately long, RUL 5.6 mm, 13% of SVL; scales on anterior and dorsal surfaces of upper arm enlarged, keeled, rhomboid, imbricate, in seven rows; scales on ventral surface of upper arm granular, homogeneous in size; scales on upper insertion of upper arm granular; scales on dorsal surface of forearm keeled, heterogeneous in size, extending to wrist; dorsal scales on hand slightly keeled; scales on palm granular; dorsal scales on fingers in a row, smooth; subdigital lamellae under fingers I–V respectively (left/right) 9/9 (3 entire + 1 divided + 1 entire), 12/12 (6 entire + 5 divided + 1 entire), 16/16 (10 entire + 5 divided + 1 entire), 22/22 (15 entire + 6 divided + 1 entire), 13/13 (6 entire + 6 decided + 1 entire); relative lengths of adpressed fingers I < V < II < III < IV; hindlimbs slender and long, fourth toe reaching the posterior edge of insertion of upper arm when hindlimb adpressed along the side of the body; HLL 25.4 mm, 59% of SVL, 129% of ALL; TFL 6.3 mm, 15% of SVL; LTL 7.9 mm, 18% of SVL; three rows of large smooth scales running beneath thigh with traces of a fourth row; two rows of enlarged keeled scales and one rows of small keeled scales on dorsal surface of thigh; granular scales homogeneous in size on rear of thigh; internal tibial scale of row one formed by enlarged and smooth tibial scale; dorsal tibial scale flat, keeled, heterogeneous in size, extending to dorsal surface of foot; scales on sole of the foot granular; dorsal scales on toes in a row, smooth; subdigital lamellae under toes I–V respectively (left/right) 9/9 (2 entire + 6 divided + 1 entire), 13/14 (7 entire + 5/6 divided + 1 entire), 18/21 (11 entire + 6 divided + 1 entire), 26/26 (2 divided + 17 entire + 6 divided + 1 entire), 18/18 (2 divided + 7 entire + 7 divided + 1 entire); basal two subdigital lamellae of toe IV and V divided; relative lengths of adpressed toes I < II < V < III < IV.

Tail original, TaL 111.3 mm, TaL/SVL ratio 259%, SVL/TaL ratio 39 %, with strongly keeled scales in 15 rows at base (fifth subcaudal scale), in 13 rows in position of the 13^th^ to 15^th^ subcaudal scales (CSR); paired vertebral series of large scales on tail extending on to hind body.

#### Coloration of holotype in life.

Dorsal surface of head, body, limbs, and tail bright brown, with a pair of strikingly yellowish-white dorsolateral lines bordered by black above and below, each beginning from the posterior margin of the most last supratemporal, running along outermost dorsal scale row, posteriorly extending to the forepart of the tail; flanks of body blackish brown with light brown marks; a pair of orange ventrolateral lines beginning from axilla, running along lower part of flanks, posteriorly extending to the groin; labial series, mental, chin-shields, granular scales on throat, collars light blue-green, posteriorly yellowish green from chest, venter, until to subcaudal region; ventral surface of limbs brown, tinged with green.

#### Coloration of holotype in preservative.

Dorsal surface of head, body, limbs and tail brown; labial series, mental, chin-shields, granular scales on throat, ventral surface of body and tail pale blue; mottles on flanks blurry, color of mottles on flanks faded; ventral surface of limbs beige; dorsolateral stripes greyish white with black-brown edges at the inner sides; color of ventrolateral stripes faded, greyish white.

#### Variations and sexual dimorphism.

Measurements, body proportions, and scale counts of the type series of *Takydromus yunkaiensis* sp. nov. are listed in Tables 2 and 4.

In the holotype SYS r001580, there are four supraciliaries on left side and two on right side, the first one longest on right side, the second supraciliary longest on left side (vs. four supraciliaries on each side, and the second one longest in the paratypes SYS r001439, 1440, 1442, 1513, 1514, 1581, 1684, 1901; three supraciliaries on each sides, and the second one longest in the paratype SYS r001507); prefrontal in contact with the anterior two supraoculars posteriorly in the holotype (vs. prefrontal only in contact with the first supraocular posteriorly on the right side of the paratype SYS r001439); three pairs of femoral pores in the holotype (vs. only two pairs present in the paratypes SYS r001513, 1514); tail relatively longer in two of the female paratypes, TaL/SVL 2.97 in SYS r001581 and 3.02 in SYS r001901 (vs. TaL/SVL 2.59 in the holotype).

*Takydromus yunkaiensis* sp. nov. exhibits noticeable sexual dimorphism:

(1) enlarged ventral scales strongly keeled in males (vs. smooth but outermost rows weakly keeled in females);

(2) dorsolateral lines strikingly yellowish-white bordered by black above and below (vs. invisible or indistinct in adult females, also in juveniles);

(3) a pair of orange ventrolateral lines present on lower flanks (vs. invisible in females, also in juveniles);

(4) flanks of body blackish brown with light brown marks in adult males (vs. absent in females).

#### Distribution and habits.

Currently, *Takydromus yunkaiensis* sp. nov. is known only from its type locality of Dawuling Forestry Station, adjacent Xianrendong Scenic Area located in the southern Yunkai Mountains in western Guangdong Province, China (Fig. 1).

**Figure 5.**
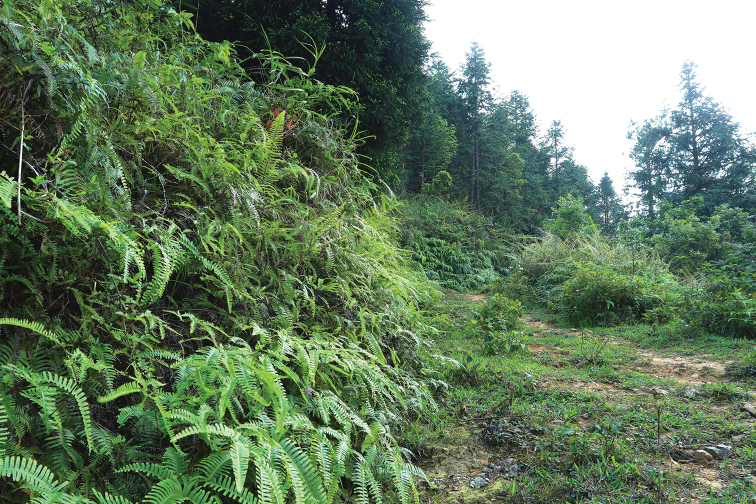
Habitat of *Takydromus yunkaiensis* sp. nov. in Yunkaishan Nature Reserve.

The diurnal species was found to be very active in daytime and rapidly escapes when disturbed, and is usually observed resting on fern leaves at night. The surrounding environment was covered by well-preserved montane evergreen broad-leaved forest or mixed forest (Fig. 5) at altitudes of 900–1600 m.

## Discussion

The description of *Takydromus yunkaiensis* sp. nov. brings the total number of species of this genus to 24, nine of which occur in mainland China. As noted, six species were recorded from Guangdong Province: *T. albomaculosus*, *T. kuehnei*, *T. septentrionalis*, *T. sexlineatus*, *T. sylvaticus*, and *Takydromus yunkaiensis* sp. nov., which further support the very high biodiversity level of the genus in southern China (Zhao et al. 1999; Lue and Lin 2008; Yang and Wang 2010; Wang et al. 2017).

Most of the early descriptions of *Takydromus* species only listed relatively limited diagnostic characteristics, resulting in considerable challenges in field identification of the species, and causing ambiguities in taxonomy. Moreover, in recent years, a number of new or cryptic species were discovered and described from southern mainland China and Taiwan Island (Lin et al. 2002; Lue and Lin 2008; Wang et al. 2017). These discoveries confirm the substantially underestimated species diversity within the tropical genus *Takydromus*, and more field research is required to increase the knowledge of the diversity.

Located in the western Guangdong Province, the Yunkai Mountains have gradually been recognized for its unique biodiversity. During herpetological surveys during the last several years, we have discovered a number of new species including some cryptic species, as well as providing new regional records of amphibians and reptiles (Yang et al. 2011; Lyu et al. 2018; Wang et al. 2018a; Wang et al. 2018b; Lyu et al. 2019), suggesting that future herpetological exploration will likely continue to yield new discoveries from the region.

## Supplementary Material

XML Treatment for Takydromus yunkaiensis

## Appendix 1

**Examined specimens**

*Takydromus albomaculosus* (*N* = 2): Chia: Guangdong Province: Tianjingshan Forest Station: SYS r001292, 1624.

*Takydromus amurensis* (*N* = 2): China: Heilongjiang Province: SYS r001635; Suifenhe City: SYS r001647.

*Takydromus wolteri* (*N* = 1): China: Heilongjiang Province: SYS r001636.

*Takydromus septentrionalis* (*N* = 25): China: Jiangxi Province: Mt. Sanqing: SYS r000179; Wuyuan County: Mt. Dazhang: SYS r000644, 653–655; Guixi City: Yangjifeng Nature Reserve: SYS r000115, 0133, 0135, 0147; Yanshan County: Wuyishan Nature Reserve: SYS r000642; Guangfeng County: Tongboshan Nature Reserve: SYS r000656, 0471, 0472, 0741, 0742, 0772; Jinggangshan City: Mt. Jinggang: SYS r000282, 1307; Zhejiang Province: Jingning County, Dongkeng: SYS r000912; Fujian Province: Wuyishan City: Sangang Village: SYS r000667, 0676, 0678; Guangdong Province: Ruyuan County: Tianjingshan Forestry Station: SYS r000929, 0930; unknown locality: SYS r000168.

*Takydromus sexlineatus* (*N* = 5): China: Guangdong Province: Fengkai County: Heishiding Nature Reserve: SYS r001335, 1336, 1337, 1552; Guangxi Zhuang Autonomous Region: Shangsi County: Shiwandashan Forest Park: SYS r000127.

*Takydromus intermedius* (*N* = 8): China: Sichuan Province: Mt. Emei: SYS r001601, 1602, CIB 3745, 3750; Guizhou Province: Libo County: Maolan Nature Reserve: SYS r000856; Hunan Province: Sangzhi County: Badagongshan Nature Reserve: SYS r001330, 1331; Guangxi Zhuang Autonomous Region: Hechi City: Jiuwanshan Nature Reserve: SYS r001553.

*Takydromus sylvaticus* (*N* = 3): China: Jiangxi Province: Guixi City: Yangjingfeng Nature Reserve: SYS r000159, 0184; Fujian Province: Shaowu City: Longhu Forestry Station: SYS r001276.

*Takydromus kuehnei* (*N* = 5): China: Jiangxi Province: Longnan County: Jiulianshan Nature Reserve: SYS r001268; Guangdong Province: Fengkai County: Heishiding Nature Reserve: SYS r000119, 0132, 1338; Renhua County: Huangshakeng Village: SYS r000206.
